# Cell Ratio-Dependent Osteoblast–Endothelial Cell Crosstalk Promoting Osteogenesis–Angiogenesis Coupling via Regulation of Microfluidic Perfusion and Paracrine Signaling

**DOI:** 10.3390/mi16050539

**Published:** 2025-04-30

**Authors:** Yuexin Wang, Shu Chen, Wenwen Fan, Sixian Zhang, Xi Chen

**Affiliations:** Key Laboratory for Ultrafine Materials of Ministry of Education, Frontiers Science Center for Materiobiology and Dynamic Chemistry, Engineering Research Center for Biomedical Materials of Ministry of Education, School of Materials Science and Engineering, East China University of Science and Technology, Shanghai 200237, China; y30220914@mail.ecust.edu.cn (Y.W.); y30220826@mail.ecust.edu.cn (S.C.); y82230286@mail.ecust.edu.cn (W.F.); y82230274@mail.ecust.edu.cn (S.Z.)

**Keywords:** osteoblast, endothelial cell, osteogenesis–angiogenesis coupling, microfluidic cell culture, organ-on-a-chip, in vitro model

## Abstract

Osteogenesis–angiogenesis coupling, a dynamic and coordinated interaction between skeletal and vascular cells, is essential for fracture healing. However, the effects of these cell ratios and their interactions under microfluidic perfusion and paracrine signaling on osteogenesis–angiogenesis coupling have rarely been reported. In this study, dynamic and static models of osteogenesis–angiogenesis coupling were developed and the osteogenic and angiogenic effects of the two models were compared. Static co-cultures of MC3T3-E1 and bEnd.3 cells in Transwell inserts showed a cell ratio-dependent reciprocal relation: a ratio of 1:1 (MC3T3-E1:bEnd.3) favored osteogenesis, whereas a ratio of 2:1 (MC3T3-E1:bEnd.3) promoted angiogenesis. On that basis, we developed an osteogenesis–angiogenesis coupling chip based on microfluidic technology. The microfluidic perfusion within the chip further enhanced the mineralizing effect of osteoblasts and the angiogenic effect of endothelial cells, respectively, and increased the secretion of vascular endothelial growth factor (VEGF) and bone morphogenetic protein-2 (BMP-2) compared to the static Transwell insert model. The results suggest that the microfluidic chip enhanced the potential of osteogenesis–angiogenesis coupling mediated by paracrine signaling. Overall, the chip is not only a powerful model for understanding bone–vascular interaction but also a scalable platform for high-throughput drug screening and personalized therapy development for fractures.

## 1. Introduction

Fracture healing is a complex dynamic physiological process involving a close spatiotemporal link between osteogenesis and angiogenesis, a link that has been termed “angiogenesis–osteogenesis coupling” [1,2,3,4,5,6]. Osteogenesis–angiogenesis coupling is characterized by coordinated crosstalk between different cellular populations, including skeletal cells (e.g., osteoblasts, osteoclasts) and vascular cells (e.g., endothelial cells, pericytes), and their microenvironment. Meanwhile, this complex cellular crosstalk could be stimulated by biochemical and biomechanical signals to achieve tissue regeneration [3,6,7]. In the cooperation among different cell populations, signal exchanges mediated by paracrine mechanisms play an important role in osteogenesis and angiogenesis [1]. During fracture healing, the relative dominance of osteogenesis and angiogenesis undergoes dynamic spatiotemporal regulation [5,8]. Early in fracture healing, active endothelial cells generate angiogenic invasion through secreted growth factors such as transforming growth factor-beta 1 (TGF-β1), TGF-β3, and matrix metalloproteinase-9 to support the recruitment, enrichment, and differentiation of osteoprogenitors [1,3,9,10], followed by enhanced osteogenesis and production of a mineralized matrix that stabilizes angiogenesis by regulating growth factors such as VEGF [11,12,13], slit guidance ligand 3 [14], and hypoxia-inducible factor-1α (HIF-1α) [15,16]. Therefore, rational regulation of the osteogenesis–angiogenesis coupling with different cell ratios mediated by paracrine mechanisms is essential for fracture healing.

Culture models are critical for studying osteogenesis–angiogenesis coupling mediated by paracrine mechanisms; however, current static models have many shortcomings. Direct co-culture systems, though operationally simple, cannot avoid direct cell contact, thus introducing cell-to-cell contact effects [17,18]. Conditioned media approaches isolate paracrine signaling but risk cytokine degradation [19]. In contrast, Transwell insert systems provide physical separation while allowing paracrine exchange, so they can be applied to study paracrine signaling [20]. Using Transwell inserts, Xu et al. demonstrated that endothelial progenitor cells co-cultured with mesenchymal stem cells (MSCs) promote osteogenic differentiation through a MAPK-dependent pathway [21]. Ge et al. showed that MSCs can mediate the differentiation of endothelial progenitor cells into endothelial cells through a paracrine mechanism [22]. However, there are fewer studies on the potential for osteogenesis–angiogenesis coupling through paracrine effects under conditions of the co-culture of osteoblasts and endothelial cells at different cell ratios.

Furthermore, this osteogenesis–angiogenesis coupling is not only mediated by the paracrine mechanisms but also modulated by mechanical stimuli in the physiological microenvironment [23]. Endothelial cells (ECs) regulate and remodel the vasculature in response to hemodynamic forces [24], and MSCs accelerate osteogenic differentiation under low levels of shear stress [25,26]. Therefore, it is necessary to introduce mechanical cues to reproduce the dynamic microenvironment of osteogenesis–angiogenesis coupling to improve the physiological relevance. A dynamic model that can provide mechanical stimulation is critical to the study of osteogenesis–angiogenesis coupling. However, animal models as a traditional dynamic research model have long lead times, high costs and ethical issues [27,28]. Therefore, a new dynamic culture model is needed. Organ-on-a-chip offers a promising solution that integrates microfluidics, 3D biomaterials, cell biology, and tunable mechanical cues [29,30]. The chip systems mimic the physiological microenvironment relevant to the human body in terms of the tissue interfaces and mechanical stimuli [31], and they allow precise control of multicellular interactions in this environment to study the kinetics of in vitro drug activity and systemic responses [32]. However, the use of organ-on-a-chip models to investigate the potential for osteogenesis–angiogenesis coupling under combined paracrine regulation and microfluidic modulation has not been studied.

In this study, we used static Transwell insert and dynamic chip models to investigate the potential of osteogenesis–angiogenesis coupling, respectively, under combined paracrine regulation and microfluidic modulation. Firstly, the osteogenesis–angiogenesis coupling was investigated by co-culture of MC3T3-E1 (an osteoblast cell line) and bEnd.3 (a vascular endothelial cell line) cells at different cell ratios in a static Transwell insert model. The ratio-dependent reciprocity between the two cells demonstrated that a 1:1 cell ratio (MC3T3-E1:bEnd.3) favored osteogenesis while a 2:1 cell ratio (MC3T3-E1:bEnd.3) enhanced angiogenesis. This cell ratio-dependent dominance of osteogenesis and angiogenesis mimics the physiological process of fracture healing, in which different stages of osteogenesis and angiogenesis occur at different rates and intensities. Then, we developed an osteogenesis–angiogenesis coupling chip and perfused it with periodically circulating microfluidic to simulate hemodynamic stimulation. The results showed that the chip model enhanced the mineralizing effect of osteoblasts as well as the expression of angiogenic markers in endothelial cells. In addition, the enzyme-linked immunosorbent assay (ELISA) experiments demonstrated that fluid shear stress promoted the secretion of VEGF and BMP-2 in co-cultured cells. Compared to the static Transwell insert model, the microfluidic chip model enhanced the potential of osteogenesis–angiogenesis coupling mediated by VEGF and BMP-2. Overall, this study not only establishes a more realistic physiological model of osteogenesis–angiogenesis coupling but also provides a foundation for developing pathological fracture models (e.g., osteoporotic fracture and aging-induced discrepant response of fracture). Furthermore, it offers a platform for high-throughput drug testing and personalized therapies targeting fracture repair.

## 2. Materials and Methods

### 2.1. Development of Chip Platforms

The chip was designed and computerized numerical control machined by Solid Works. The chip consisted of three parts: polydimethylsiloxane (PDMS) top substrates, bottom substrates and a polyester (PET) membrane. The top and bottom basal rectangular channels were separated by a PET membrane (0.4 µm pore size) to allow simultaneous seeding of multiple cell types in different channels and liquid perfusion co-culture without direct contact (Figure S1A) [33,34]. In addition, holes were punched at the same locations on the substrate and the PET membrane to serve as inlets and outlets for the microfluidics. Finally, it was reinforced with polymethyl methacrylate (PMMA) sheets (Figure S1B).

The chips were fabricated by the molding method, in which the PDMS adhesive and curing agent (SYLGARDTM 184 Silicone Elastomer, Dow Corning) were poured into the molds (Figure S1C) at a mass ratio of 10:1, and then the top and bottom substrates were obtained through the steps of defoaming, curing, peeling and cutting. The PET membrane was then immersed in a 5% (*v*/*v*) solution of 3-aminopropyltriethoxysilane (APTES, Sigma-Aldrich) for 30 min, washed with distilled water and dried at room temperature, and then the PET membrane and top and bottom layers of PDMS were treated with oxygen plasma (Plasma PTL-VM500) using the established treatment parameters (20 SCCM O_2_, 100 W, 60 s). Finally, the parts were assembled from top to bottom, picked up with tweezers and placed under a microscope for aligned conformational contact, and finally, baked in an oven at 60 °C for 24 h.

### 2.2. Flow Rate Analysis

The fluid shear stress is a key parameter of the mechanical stimulation that keeps the co-cultured cells in a dynamic microenvironment close to human physiology. The fluid shear stress on the chips can be calculated according to Equation (1) [35].

(1) $$T=\frac{6\mu Q}{h^{2}w}$$

where *T*: fluid shear stress; *Q*: flow rate; *μ*: fluid viscosity, relative to the medium; *h*: channel height; and *w*: channel width.

COMSOL 6.2 Multiphysics was used to simulate the forces generated in the chip. The results show that the fluid shear stress inside the channel (red border) was between 0.8 and 1.0 Pa, which is within the range of human physiological pressure [36]. With reference to the formulas and simulation, the width of the channel was set at 0.9 mm, the height of the upper channel at 1 mm, the height of the lower channel at 0.2 mm, the angles of entry and exit at 60°, and the flow rate at 10 µL/min in the subsequent experiments to simulate the magnitude of shear stress of osteoblasts and endothelial cells in the human body.

### 2.3. Cell Culture

Mouse osteoblast precursor cells MC3T3-E1 (Procell, Wuhan, China) and mouse microvascular endothelial cells bEnd.3 (Procell, Wuhan, China) exhibit an osteoblast phenotype and a vascular cell phenotype, respectively. The MC3T3-E1 and bEnd.3 cells were cultured in MEM Alpha Modification (α-MEM, Gibco, Grand Island, NY, USA) medium and Dulbecco’s modified Eagle’s medium (DMEM, Gibco, Grand Island, NY, USA) containing 10% fetal bovine serum (FBS, Gibco, Grand Island, NY, USA) and 1% penicillin–streptomycin (P/S, Gibco, Waltham, MA, USA), respectively. The MC3T3-E1 and bEnd.3 cells were incubated in an incubator (37 °C, 5% CO_2_) and fluid exchange was performed every 2 days. When the confluence of the cells reached about 80–90%, trypsin (Gibco, Waltham, MA, USA) was used to digest the cells. Cells grown for 3–6 generations in the logarithmic growth phase were selected for culture on the Transwell insert (0.4 µm pore size, Corning, NY, USA) and chip.

### 2.4. Co-Culture

**Figure 1:**
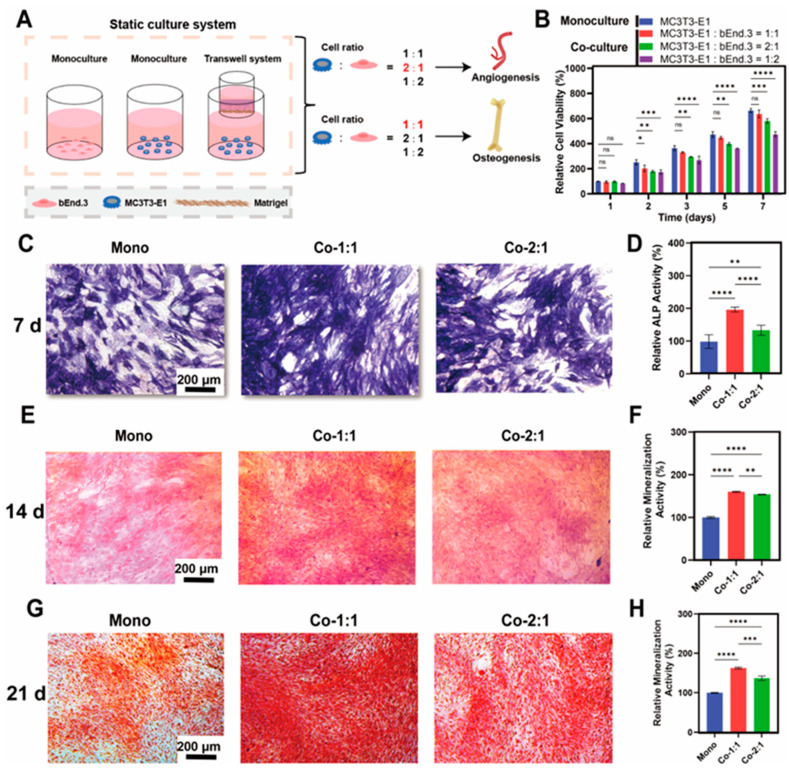
Osteogenic benefits of MC3T3-E1 and bEnd.3 cells co-cultured at different cell ratios. (**A**) Schematic of the static indirect co-culture system. (**B**) Effect of co-culture on MC3T3-E1 proliferation as determined by CCK-8. (**C**) Representative images of 7-day ALP staining. (**D**) ALP activity assay. (**E**) Representative images of 14-day ARS staining. (**F**) Fourteen-day ARS quantification. (**G**) Representative images of 21-day ARS staining. (**H**) Twenty-one-day ARS quantification. Mono: monoculture, Co-1:1: MC3T3-E1:bEnd.3 = 1:1, Co-2:1: MC3T3-E1:bEnd.3 = 2:1. * *p* < 0.05, ** *p* < 0.01, *** *p* < 0.001, **** *p* < 0.0001, ns. not significant. *n* ≥ 3, the *n* represents the number of independent samples.

The cells are divided into three groups: MC3T3-E1 monoculture, bEnd.3 monoculture and co-culture (MC3T3-E1 and bEnd.3 co-cultured in 1:1, 1:2, and 2:1 ratios). The bEnd.3 inoculation chamber was added to the 6-well plate inoculated with MC3T3-E1 cells to establish the cell co-culture system (Figure 1A). The respective growth medium was used for both. When the cell fusion reached 70–80%, the medium in the lower chamber was replaced with osteogenic induction medium (OriCell, Guangzhou, China) and the incubation was continued in an incubator (37 °C, 5% CO_2_).

### 2.5. Cell Culture on Chip

The chip was placed on ice, rinsed three times with pre-cooled phosphate buffer, and Matrigel (Corning, NY, USA) medium was applied to the chip. All the operations were performed on ice, and then the chip was placed in an incubator (37 °C, 5% CO_2_, 30 min), the cell density was adjusted to 1×10^6^ cells/mL, and the cell suspension was injected into the channel with a 200 µL syringe tip. The microfluidic chip was continuously cultured in the incubator for 1 d, 7 d, and 14 d, and then the cell morphology was observed.

### 2.6. Cell Proliferation Assay

The Cell Counting Kit-8 (CCK-8, Beyotime, Shanghai, China) was used to evaluate the effect of co-culture on the proliferation of MC3T3-E1 and bEnd.3 cells. Different ratios were used to co-culture the two cell types for 1 d, 3 d, 5 d and 7 d. The CCK-8 solution was added and incubated for 2 h, then the absorbance was measured at 450 nm.

### 2.7. Osteogenic Differentiation Assay

To determine the effects of the co-culture conditions and microfluidic chips on the osteogenic differentiation of MC3T3-E1 cells, alkaline phosphatase (ALP) and mineral deposition were assayed. ALP staining and an activity assay were performed according to the manufacturer’s instructions (Beyotime, Shanghai, China) after 7 days of osteogenic induction. On day 14 and 21 of differentiation, Alizarin Red S (ARS, OriCell, Shanghai, China) staining was performed to assess the mineral deposition. For quantitative analysis of the mineralization, the deposited calcium was eluted with 10% (*w*/*v*) cetylpyridinium chloride (CPC, Sigma-Aldrich, St. Louis, MO, USA) and the absorbance was measured at 565 nm.

### 2.8. In Vitro Angiogenesis-Related Assays

Tube formation assay: bEnd.3 cells (1 × 10^5^ cells/well) were inoculated into 24-well plates coated with Matrigel (Corning, NY, USA) and incubated in different conditioned media for 5 h. The branch length and branch number were then measured to assess the tube formation ability.

Transwell migration assay: bEnd.3 cells (2 × 10^4^ cells/well) were seeded in the upper chamber of 24-well, 8 µm pore size transwell plates (Corning, NY, USA) and then cultured with different ratios of osteoblasts in the lower chamber for 16 h. Unmigrated cells remaining in the upper chamber were then removed with a cotton swab, while migrated cells passing through the membrane pores were fixed with 4% (*w*/*v*) paraformaldehyde (PFA, Servicebio, Wuhan, China) for 15 min and stained with 0.1% (*w*/*v*) crystal violet (Solarbio, Beijing, China) for 5 min for photography, after which the crystal violet was completely eluted by destaining with 33% acetic acid and the absorbance was measured at 565 nm.

Scratch wound assay: bEnd.3 cells were inoculated into 6-well plates and grown to confluence. The confluent cell layer was then scratched with a sterile pipette tip. Images of the wound were taken immediately and 24 h later. The images were analyzed using ImageJ software.

### 2.9. Enzyme-Linked Immunosorbent Assay

The VEGF and BMP-2 secretion was assessed using the mouse VEGF ELISA kit (NeoBiosccience, Shenzhen, China) and the BMP-2 ELISA kit (Neobiosccience, Shenzhen, China). The supernatants from the monoculture, co-culture and chip were collected on day 7 for assay. All the procedures were performed according to the manufacturer’s instructions.

### 2.10. Quantitative Real-Time Polymerase Chain Reaction Analysis

The quantitative reverse transcription polymerase chain reaction (RT-qPCR) was used to assess the mRNA expression levels of ALP, VEGF, vascular endothelial growth factor A (VEGF-A), vascular endothelial growth factor receptor 1 (VEGFR1), vascular endothelial growth factor receptor 2 (VEGFR2), Runt-related transcription factor 2 (Runx2), collagen type I (COL1), Runt-related transcription factor 2 (Runx2), bone morphogenetic protein receptor type 2 (BMPR2), platelet endothelial cell adhesion molecule-1 (PECAM-1/CD31) and hypoxia inducible factor-1α (HIF-1α) in the different experimental groups. The total RNA was isolated using TRIzol reagent and reverse transcribed into cDNA using the PrimeScript™ RT Reagent Kit (Takara, Otsu, Shiga, Japan). Finally, the qPCR reaction was performed using the TB Green^®^ Premix Ex Taq™ II Reagent Kit (Takara, Japan) on a CFX96 PCR detection system (Bio-Rad, Hercules, CA, USA). Each sample had three replicate wells to ensure the reliability of the experimental results. The primer sequences are listed in Tables S1 and S2. The 2−^ΔΔCt^ relative expression method was used for the data analysis.

### 2.11. Immunofluorescence Staining

The cells were washed 3 times with PBS, fixed with 4% PFA for 15 min at room temperature, permeabilized with 0.5% Triton X-100 for 15 min and blocked with 3% bovine serum albumin for 30 min. The cells were incubated with primary antibody overnight at 4 °C. After three washes with Dulbecco’s phosphate-buffered saline to remove the primary antibody, the cells were incubated with fluorescein isothiocyanate (FITC)-conjugated secondary antibody at 37 °C for 1.5 h and the nuclei were labelled with 4′,6-diamidino-2′-phenylindole (DAPI) staining solution. Fluorescence images were captured using a digital imaging system (APX100, Olympus, Shinjuku, Tokyo, Japan). The antibodies used are listed in Table S3, and the images were processed using ImageJ.

### 2.12. Statistical Analysis

Data are expressed as the mean ± standard deviation (SD), and at least three parallel samples were set up for each experiment (*n* ≥ 3). The trend of all the data was confirmed by 3 independent replicated experiments. Statistical analysis was performed with GraphPad Prism 10.0 statistical software. Quantitation was performed using ImageJ 1.54f software. The t-test and one-way analysis of variance (ANOVA) were used for statistical analysis, and *p* < 0.05 was considered statistically significant; * *p* < 0.05, ** *p* < 0.01, *** *p* < 0.001, **** *p* < 0.0001, ns. not significant.

## 3. Results

### 3.1. Osteogenic Benefits of MC3T3-E1 and bEnd.3 Cells Co-Cultured at Different Cell Ratios

To investigate the effects of osteoblast–endothelial cell interactions mediated by paracrine mechanisms under co-culture at different cell ratios, we established an indirect co-culture system of MC3T3-E1 and bEnd.3 cells using Transwell inserts (Figure 1A). First, cell viability assays were performed to evaluate the effect of bEnd.3 cells on the proliferation of MC3T3-E1 cells in co-culture groups with different cell ratios. CCK-8 assays showed that there was a temporal increase in the MC3T3-E1 viability in all the groups, with no significant differences observed between the monoculture group and the ratio of 1:1 co-culture group of MC3T3-E1 and bEnd.3 cells in terms of survival over a 7-day period (Figure 1B). Notably, the ratio of the 2:1 (MC3T3-E1:bEnd.3) co-culture group showed a marginal reduction in viability (13 ± 2%), while the ratio of the 1:2 (MC3T3-E1:bEnd.3) co-culture group showed a more pronounced inhibition (29 ± 2%). Meanwhile, a ratio of 1:1 mixed medium of MC3T3-E1 and bEnd.3 cell media was used to culture MC3T3-E1 cells, and the results showed that this ratio of mixed medium had a negligible effect on the proliferation of MC3T3-E1 cells (Figure S3A). The results indicated that co-culture with bEnd.3 cells did not promote the proliferation of MC3T3-E1 cells. This may be due to the competition for nutrients with bEnd.3 cells under co-culture conditions. Based on these results, subsequent experiments focused on the 1:1 and 2:1 (MC3T3-E1:bEnd.3) co-culture groups.

To evaluate the effect of bEnd.3 cells on MC3T3-E1 osteogenesis in co-culture groups with different cell ratios, ALP and ARS staining experiments were performed to examine the differentiation and mineralization of MC3T3-E1 cells, respectively. The ALP staining and activity measurements after co-culturing for 7 days (Figure 1C,D) showed that both cell ratio co-culture groups increased the ALP viability of the MC3T3-E1 cells compared to the monoculture group, but the MC3T3-E1 cells in the 1:1 co-culture group exhibited the highest ALP activity (96% increased vs. monoculture group). In addition, ARS staining and calcium quantification after 14 days of co-culture showed that both bEnd.3 cells in the co-culture groups promoted calcium nodule deposition in MC3T3-E1 cells compared to the monoculture, with the best effect observed in the 1:1 co-culture group (Figure 1E,F). Meanwhile, ARS staining and calcium quantification after 21 days of co-culture further confirmed this result (Figure 1G,H). Taken together, these data suggest that a 1:1 ratio of MC3T3-E1 and bEnd.3 cells was more optimal for osteogenesis under a Transwell co-culture model.

### 3.2. Expression of Osteogenic Markers in MC3T3-E1 and bEnd.3 Cells Co-Cultured at Different Cell Ratios

**Figure 2:**
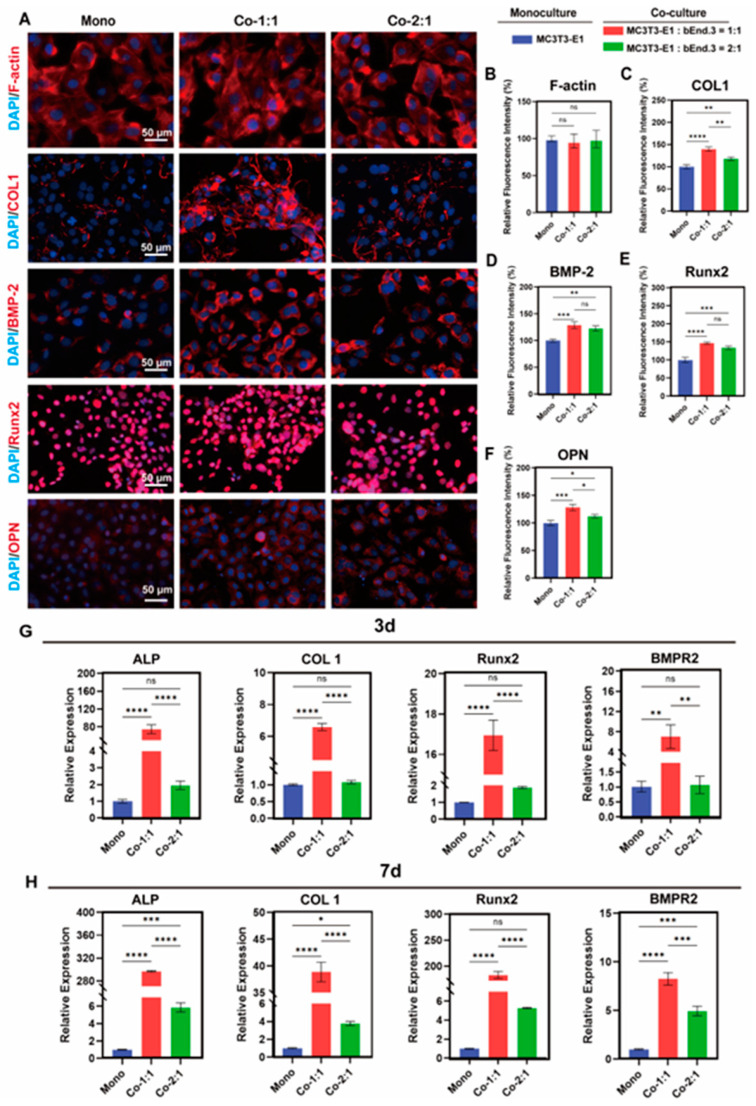
Expression of osteogenic markers in MC3T3-E1 and bEnd.3 cells co-cultured at different cell ratios. (**A**) Filamentous actin (F-actin) staining and immunofluorescence staining of COL1, BMP-2, Runx2 and OPN in MC3T3-E1 after 3 days of co-culture. (**B**–**F**) Quantitative results of F-actin, COL1, BMP-2, Runx2 and OPN expression, respectively. (**G**) Expression levels of osteogenesis-related genes ALP, COL1, Runx2, BMPR2 after 3 days of co-culture. (**H**) Expression levels of osteogenesis-related genes ALP, COL1, Runx2, BMPR2 after 7 days of co-culture. Mono: monoculture, Co-1:1: MC3T3-E1:bEnd.3 = 1:1, Co-2:1: MC3T3-E1:bEnd.3 = 2:1. * *p* < 0.05, ** *p* < 0.01, *** *p* < 0.001, **** *p* < 0.0001, ns. not significant. *n* ≥ 3, the *n* represents the number of independent samples.

To further illustrate the effect of co-culture on osteogenesis, we evaluated the expression of angiogenesis-related genes and proteins in MC3T3-E1 cells in co-culture groups with different cell ratios. After 3 days of co-culture, immunofluorescence analysis revealed enhanced expression of osteogenic markers, including COL1, BMP-2, Runx2 and osteopontin (OPN), in the MC3T3-E1 cells in the 1:1 co-culture group compared to the monoculture group (Figure 2A–F). In addition, the RT-qPCR results after 3 days of co-culture showed that the expression levels of ALP, COL1, Runx2 and BMPR2 were increased in the MC3T3-E1 cells in the 1:1 co-culture group compared to the monoculture group (Figure 2G). This result was further confirmed by RT-qPCR after 7 days of co-culture, and the up-regulation of these genes was more pronounced in the MC3T3-E1 cells in the 1:1 co-culture group compared to co-culture after 3 days (Figure 2H). Notably, the expression of VEGF-A and VEGFR2 was also increased in the MC3T3-E1 cells in the 1:1 co-culture group compared to the monoculture group after 7 days of co-culture (Figure S4). Taken together, these results demonstrate that the 1:1 co-culture of MC3T3-E1 and bEnd.3 cells increased the expression of osteogenesis-related markers in MC3T3-E1 cells, providing a basis for the potential of enhanced osteogenesis–angiogenesis coupling.

### 3.3. Angiogenic Benefits of MC3T3-E1 and bEnd.3 Cells Co-Cultured at Different Cell Ratios

**Figure 3:**
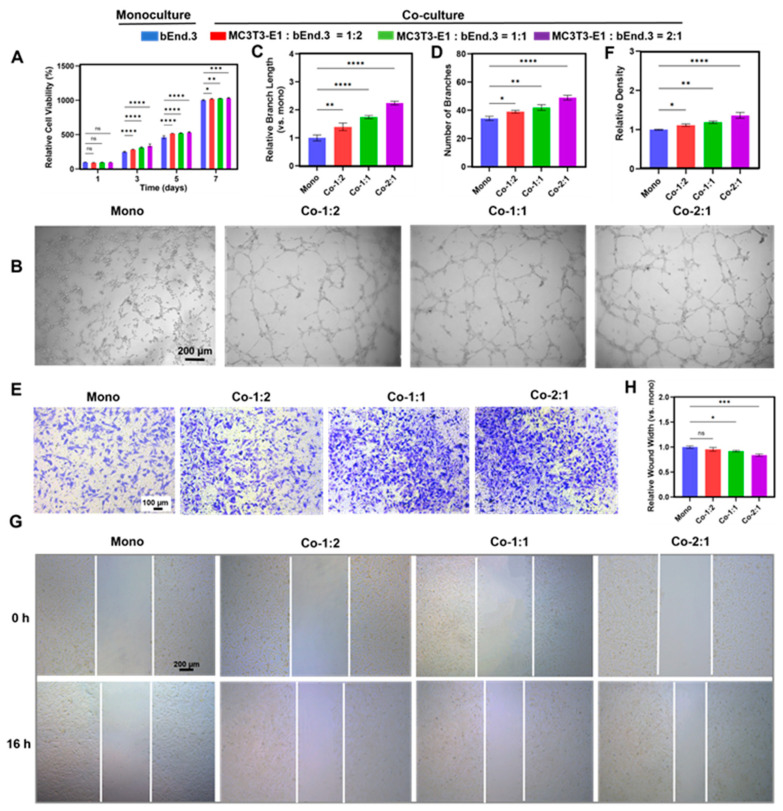
Angiogenic benefits of MC3T3-E1 and bEnd.3 cells co-cultured at different cell ratios. (**A**) Effect of co-culture with different cell ratios on bEnd.3 cell proliferation. (**B**) Tube formation assay of bEnd.3 cells under different ratios of co-culture conditions. (**C**) Quantification of the tube formation assay of the branch length. (**D**) Quantification of the tube formation assay of the branch number. (**E**) Transwell migration assay. (**F**) Quantification of the Transwell migration assays. (**G**) Scratch wound assay. (**H**) Quantification of the scratch wound assay. Mono: monoculture, Co-1:2: MC3T3-E1:bEnd.3 = 1:2, Co-1:1: MC3T3-E1:bEnd.3 = 1:1, Co-2:1: MC3T3-E1:bEnd.3 = 2:1. * *p* < 0.05, ** *p* < 0.01, *** *p* < 0.001, **** *p* < 0.0001, ns. not significant. *n* ≥ 3, the *n* represents the number of independent samples.

To evaluate the effect of MC3T3-E1 cells on the angiogenesis of bEnd.3 cells in co-culture groups with different cell ratios, angiogenesis-related experiments were performed. The CCK-8 assay showed that all the co-culture groups promoted the proliferation of bEnd.3 cells compared to the monoculture group on days 3, 5, and 7 (Figure 3A), and the promotion effect increased with the decrease of the proportion of bEnd.3 cells in the co-culture groups, which were MC3T3-E1:bEnd.3 = 1:2 (67%), 1:1 (50%), 2:1 (33%), respectively. The best effect was obtained in the 2:1 co-culture group. Meanwhile, a ratio of 2:1 mixed medium of MC3T3-E1 and bEnd.3 cell media was used to culture bEnd.3 cells, and the results showed that this ratio of mixed medium had a negligible effect on the proliferation of bEnd.3 cells (Figure S3B). Compared to the monoculture group, co-culture with different cell ratios promoted the bEnd.3 cells to form more capillary-like structures (Figure 3B), which manifested as an increase in the branch length and branch number (Figure 3C,D), and the promotion effect was negatively correlated with the percentage of bEnd.3 cells, and the best effect was seen in the 2:1 co-culture group. In addition, the Transwell inserts migration assay showed that co-culture could promote the migration of bEnd.3 cells, and the promotion effect was negatively correlated with the percentage of bEnd.3 cells, and the best effect was observed in the 2:1 co-culture group (Figure 3E,F). The scratch wound assay further confirmed this result (Figure 3G,H), but the 1:2 co-culture group was not significantly different from the monoculture. This result demonstrated that osteoblasts positively regulate endothelial cell migration and angiogenesis, which is consistent with previous reports [7,13,37]. Therefore, the 1:1 and 2:1 co-culture groups were selected for subsequent experiments. These results suggest that a 2:1 co-culture of MC3T3-E1 and bEnd.3 cells was more favorable for angiogenesis.

### 3.4. Expression of Angiogenic Markers in MC3T3-E1 and bEnd.3 Cells Co-Cultured at Different Cell Ratios

**Figure 4:**
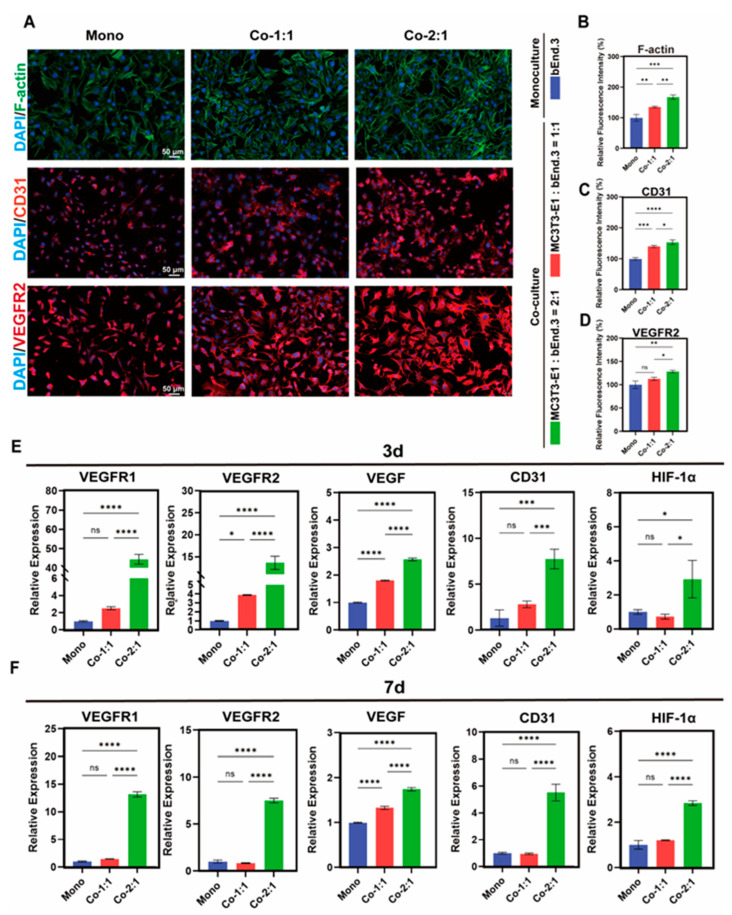
Expression of angiogenic markers in MC3T3-E1 and bEnd.3 cells co-cultured at different cell ratios. (**A**) F-actin staining and immunofluorescence staining for CD31 and VEGFR2 in bEnd.3 cells after 3 days of co-culture. (**B**–**D**) Quantitative results of F-actin, CD31 and VEGFR2. (**E**) Expression levels of angiogenesis-related genes VEGFR1, VEGFR2, VEGF, CD31 and HIF-1α in bEnd.3 cells after 3 days of co-culture. (**F**) Expression levels of angiogenesis-related genes VEGFR1, VEGFR2, VEGF, CD31 and HIF-1α in bEnd.3 cells after 7 days of co-culture. Mono: monoculture, Co-1:1: MC3T3-E1:bEnd.3 = 1:1, Co-2:1: MC3T3-E1:bEnd.3 = 2:1. * *p* < 0.05, ** *p* < 0.01, *** *p* < 0.001, **** *p* < 0.0001, ns. not significant. *n* ≥ 3, the *n* represents the number of independent samples.

To further illustrate the effect of co-culture on angiogenesis, we assessed the expression of angiogenesis-related genes and proteins in bEnd.3 cells in co-culture groups with different cell ratios. Immunofluorescence staining showed that the co-culture groups with different cell ratios promoted the expression of CD31 in the bEnd.3 cells compared to the monoculture group, with the best effect seen in the bEnd.3 cells in the 2:1 co-culture MC3T3-E1 and bEnd.3 group. Meanwhile, the VEGFR2 expression was also highest in the bEnd.3 cells in the 2:1 co-culture group (Figure 4A–D). The RT-qPCR results showed that after 3 days of co-culture, the expression of VEGFR1, VEGFR2, VEGF, CD31 and HIF-1α was significantly up-regulated in the bEnd.3 cells in the 2:1 co-culture group compared to the monoculture group (Figure 4E). In addition, all of these genes were up-regulated in the 2:1 co-culture group on day 7 compared to the monoculture group, but to a lesser extent than on day 3 (Figure 4F). It was hypothesized that under co-culture conditions, bEnd.3 cells regulated the function of both cell types through the secretion of HIF-1α and VEGF [13,15], and that the promotion of angiogenesis occurred mainly in the early phase of co-culture and diminished over time [6,15]. These results suggest that the 2:1 co-culture of MC3T3-E1 and bEnd.3 cells increased the expression of angiogenesis-related markers in the bEnd.3 cells, providing a basis for the potential of enhanced osteogenesis–angiogenesis coupling.

### 3.5. Osteogenesis–Angiogenesis Coupling on a Chip

**Figure 5:**
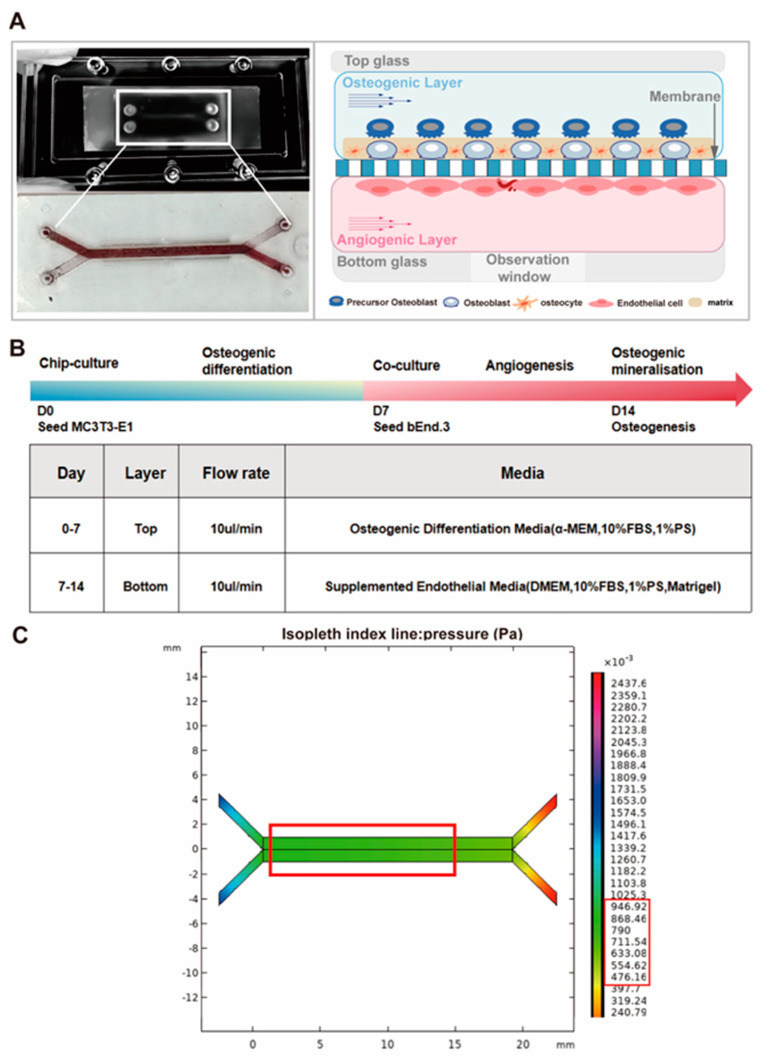
Osteogenesis–angiogenesis coupling on a chip. (**A**) Physical and schematic diagrams of the osteogenesis–angiogenesis coupling chip. (**B**) Incubation schedule. (**C**) Simulation results of the shear stress generated within the chip.

To study the interaction effects of osteogenesis and angiogenesis under dynamic stimulation, we developed an organ-on-a-chip model mimicking osteogenesis–angiogenesis coupling based on microfluidic technology consisting of two channels separated by a PET membrane for the indirect co-culture of cells. In addition, the chip was reinforced by a housing frame with a viewing window for prolonged and stable microfluidic perfusion (Figure 5A). MC3T3-E1 cells were inoculated into the upper channel to establish a bone matrix deposition layer. These cells were cultured in osteoblast differentiation medium for 7 days and perfused for 6 h of microfluidic cycling at a flow rate of 10 µL/min per day. The bEnd.3 cells were then inoculated in the lower channel for dynamic osteoblast–endothelial cell co-culture. The ratio of co-cultured cells was consistent with the optimal ratio for static co-culture. After 7 days of dynamic co-culture, the chip was harvested on day 14 for analysis (Figure 5B). Fluid flow in the human bone microenvironment can be induced by mechanical loading, muscle contraction, blood pressure, and lymphatic drainage, resulting in physiological levels of fluid shear stress [38]. Meanwhile, osteoblasts and endothelial cells in the bone microenvironment can sense, transduce, and respond to mechanical stimuli through cellular structures such as the cytoskeleton, connexins, and matrix strain [39,40]. This provides theoretical support to explain the mechanisms by which the microfluidic chip model influences coupling effects. The dynamic chip model introduced mechanical cues for microfluidic stimulation to improve the physiological relevance. Furthermore, we deduced from the numerical simulations that the shear stress values within the chip channel were between 0.8 and 1.0 Pa, which met the mechanical stress requirements that MC3T3-E1 and bEnd.3 cells must withstand in a physiological environment (Figure 5C). This is also consistent with the results of Weinbaum’s numerical simulations, in which the fluid shear stress felt by osteocytes in their proteoglycan-filled lumen ranged from 0.8 to 3.0 Pa [36].

### 3.6. The Osteogenic Effect of Osteogenesis–Angiogenesis Coupling in a Microfluidic Chip

**Figure 6:**
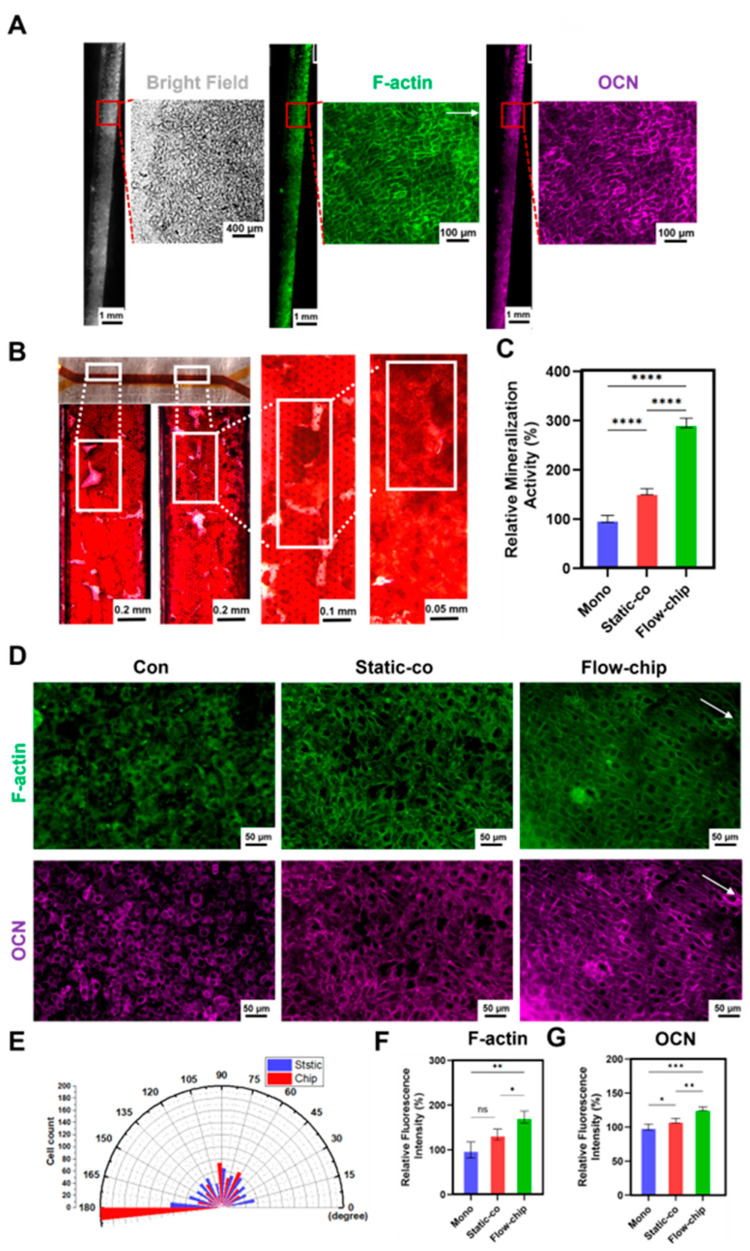
The osteogenic effect of osteogenesis–angiogenesis coupling in a microfluidic chip. (**A**) Brightfield and F-actin and OCN staining of MC3T3-E1 cells after 14 days culture on the chip. (**B**) Representative images of ARS staining. (**C**) Quantification of ARS staining. (**D**) Expression levels of F-actin and OCN after 3 days of co-culture under different conditions. (**E**) Analysis of the cytoskeleton orientation. (**F**,**G**) Quantification of F-actin and OCN. The white arrows represent the fluid direction. * *p* < 0.05, ** *p* < 0.01, *** *p* < 0.001, **** *p* < 0.0001, ns. not significant. *n* ≥ 3, the *n* represents the number of independent samples.

To demonstrate that the chip can perfuse cells at a microfluidic rate of 10 µL/min for a long period of time, we recorded the growth state of the MC3T3-E1 cells within the chip using a microscope. Brightfield images of the MC3T3-E1 cells at different time points showed that the cells remained morphologically normal throughout the 14 days of culture on the chip (Figure S5). In addition, we demonstrated the effects on the growth status and mineralized matrix formation of the MC3T3-E1 cells after 14 days of culture on the chip by immunofluorescence and ARS staining. Brightfield images showed that the MC3T3-E1 cells grew vigorously, completely occupying the channel membrane and surrounding the channel wall. In addition, intra-channel F-actin and osteocalcin (OCN) staining showed that the MC3T3-E1 cells exhibited directional growth (fluid direction, 180°) and effective differentiation upon microfluidic stimulation on the chip (Figure 6A). ARS staining showed a large number of calcium nodules (Figure 6B), and the quantitative results showed that the formation of calcium nodules on the chip was 1.90 times higher than that of the static co-culture (Figure 6C). This result indicates that the chip promoted osteogenic mineralization of co-cultured MC3T3-E1 cells. Meanwhile, we hypothesized that mechanical stimulation could affect mineralization by inducing cytoskeletal rearrangements of the osteoblasts [41]. To further illustrate the effect of the microfluidic chip on MC3T3-E1 osteogenic differentiation, we performed immunofluorescence staining of the MC3T3-E1 cells in different models. F-actin and OCN staining (Figure 6D) showed that the cytoskeleton of the MC3T3-E1 cells was significantly oriented along the microfluidic direction (180°), up to more than 75% after 3 days of co-culture on the chip (Figure 6E). The quantitative results showed that the expression of F-actin and OCN on the chip was 1.29 times (Figure 6F) and 1.27 times (Figure 6G) higher than that of the static co-culture, respectively. This result indicates that the microfluidic chip promoted the directional stretching of the MC3T3-E1 cytoskeleton and the expression of osteogenic markers. In a word, these results confirm that the microfluidic chip enhanced the osteogenic effect of osteogenesis–angiogenesis.

### 3.7. The Angiogenic Effect of Osteogenesis–Angiogenesis Coupling in a Microfluidic Chip

**Figure 7:**
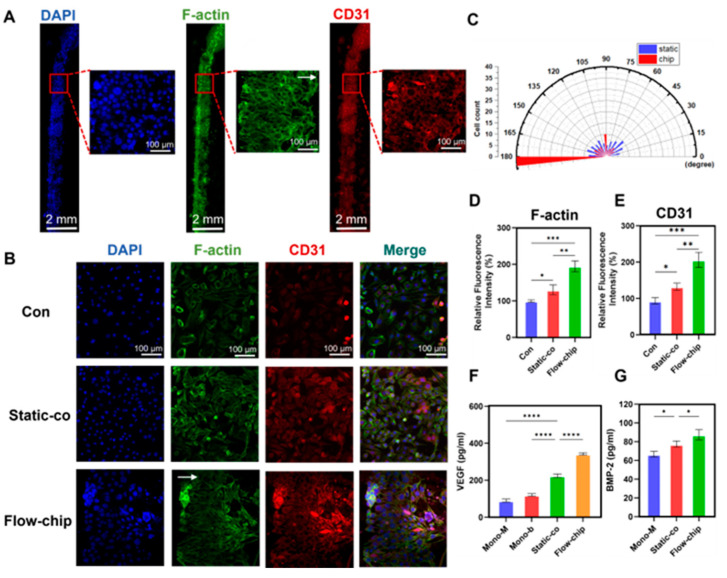
The angiogenic effect of osteogenesis–angiogenesis coupling in a microfluidic chip. (**A**) Staining of F-actin and CD31 in bEnd.3 cells after 7 days of culture on the chip. (**B**) Expression levels of F-actin and CD31 after 3 days of co-culture in different conditions. (**C**) Cytoskeleton orientation analysis. (**D**,**E**) Quantification of F-actin and CD31. (**F**,**G**) VEGF and BMP-2 secretion levels in different culture conditions. The white arrows represent the fluid direction. Mono-M: Mono-MC3T3-E1, Mono-b: Mono-bEnd.3. * *p* < 0.05, ** *p* < 0.01, *** *p* < 0.001, **** *p* < 0.0001, ns. not significant. *n* ≥ 3, the *n* represents the number of independent samples.

To demonstrate that the chip can perfuse cells at a microfluidic rate of 10 µL/min for a long period of time, we recorded the growth state of the bEnd.3 cells within the chip using a microscope. The brightfield images of the bEnd.3 cells cultured on the chip showed that the cells remained morphologically normal for 14 days (Figure S5). In addition, we demonstrated the effects on the bEnd.3 cell growth status and angiogenesis by immunofluorescence staining after 14 days of culture on the chip. Nuclear staining showed that the bEnd.3 cells grew vigorously, occupying the channel membrane and surrounding the channel wall (Figure 7A). F-actin and CD31 staining showed that the bEnd.3 cells grew directionally (fluid direction, 180°) in response to microfluidic stimulation, resulting in the formation of a functional endothelial layer. It was suggested that shear stress drives the rearrangement of the bEnd.3 cytoskeleton, presumably affecting angiogenesis [41]. To further illustrate the effect of microfluidic chips on bEnd.3 cell angiogenesis, we performed immunofluorescence staining of the bEnd.3 cells in different models. After 3 days of co-culture, F-actin and CD31 staining of the bEnd.3 cells (Figure 7B) showed that the bEnd.3 cells were highly aligned along the microfluidic direction, up to more than 75% (Figure 7C). The quantitative results showed that the expression of F-actin and CD31 on the chip was 1.49 times and 1.55 times higher than that of the static co-culture, respectively (Figure 7D,E). These results suggest that the chips enhanced the angiogenic effect of osteogenesis–angiogenesis.

In addition, the cytokines were quantitatively assessed by ELISA to explore the cellular communication mediated by paracrine mechanisms under co-culture conditions. The static co-culture model showed significantly increased secretion of VEGF and BMP-2 compared to the monoculture. Mechanical stimulation further enhanced this secretion capacity, with the chip model showing 1.52 times higher VEGF levels and 1.14 times higher BMP-2 levels compared to the static co-culture model (Figure 7F,G). The mechano-enhanced paracrine profile suggested that fluid shear stress optimized VEGF- and BMP-2-mediated osteogenesis–angiogenesis coupling. Although the dual role of VEGF in osteogenesis and angiogenesis was well established, the endothelial regulatory role of BMP-2 remained underexplored. Therefore, we demonstrated the effect of BMP-2 on bEnd.3 cells through a series of experiments. The CCK-8 assay showed that 500 ng/mL BMP-2 treatment promoted the proliferation of bEnd.3 cells (Figure S6A), and the ability of BMP-2 to promote migration was also demonstrated by the Transwell inserts (Figure S6B,C) and scratch assays (Figure S6D,E). Furthermore, immunofluorescence staining confirmed the BMPR2 expression in the bEnd.3 cells (Figure S6F,G). BMP-2 stimulation resulted in the up-regulation of CD31 expression (Figure S6H), and co-culture resulted in increased BMPR2 levels in the bEnd.3 cells (Figure S6I). Together with the positive role of BMP-2 in osteogenesis, these results suggest a dual role for BMP-2 in promoting osteogenesis and angiogenesis.

## 4. Discussion

**Figure 8:**
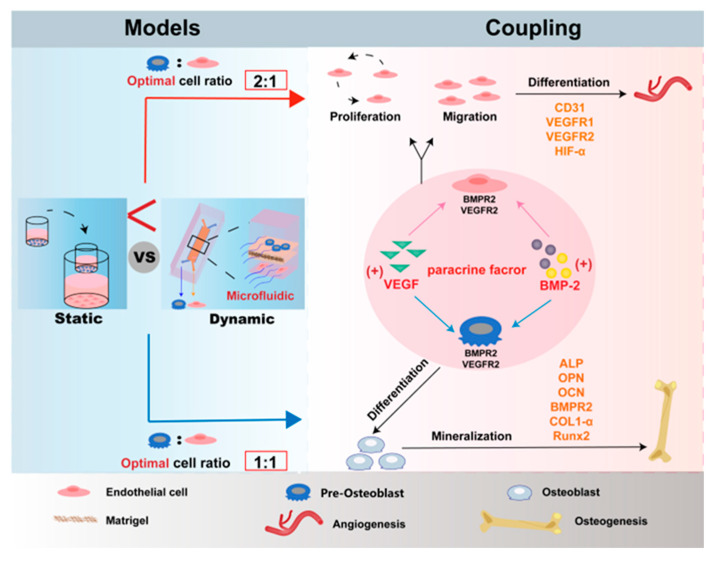
Scheme of an in vitro culture model of osteogenesis–angiogenesis coupling. Microfluidic chips enhance the potential for VEGF- and BMP-2-mediated osteogenesis–angiogenesis coupling.

Key to fracture healing is the timely appearance of blood vessels in the healing tissue, which indicates that angiogenesis and osteogenesis activity are closely related [4]. Angiogenesis not only provides oxygen and nutrients but also secretes growth factors to create a microenvironment suitable for the survival of MSCs and pre-osteoblasts, thereby promoting osteogenic differentiation [3,7,42,43]. The enhancement of osteogenic activity, in turn, further stabilizes angiogenesis [7,12,13,42]. Therefore, osteogenesis–angiogenesis coupling has attracted much attention from researchers as being key to the development of fracture healing. Cellular crosstalk between osteoblasts and vascular endothelial cells regulates the processes of osteogenesis and angiogenesis [44,45]. However, the potential for osteogenesis–angiogenesis coupling under combined mechanical and paracrine regulation has rarely been studied [46,47]. In this study, we established a static model of co-cultured MC3T3-E1 and bEnd.3 cells at different cell ratios in Transwell inserts. In the static model, we investigated the effect of bEnd.3 cells in co-culture with MC3T3-E1 cells on osteogenesis and angiogenesis. The results showed that a 1:1 (MC3T3-E1:bEnd.3) co-culture ratio optimally recapitulates the enhanced osteogenic activity (Figure 1 and Figure 2), closely mimicking the intensified capacity for osteogenesis observed in the mature phase (mid-to-late stages) of fracture repair. Meanwhile, a 2:1 (MC3T3-E1:bEnd.3) co-culture ratio optimally recapitulates the enhanced angiogenic activity (Figure 3 and Figure 4), closely mimicking the increased capacity for angiogenesis observed in the early phase of fracture repair. The reciprocal relation was recapitulated using the ratio-dependent co-culture system, demonstrating that the disproportionate dominance of either the angiogenesis or osteogenesis process impacted the efficiency of fracture healing. In addition, VEGF and BMP-2 were increased under the co-culture model (Figure 7F,G), suggesting that VEGF and BMP-2 mediated the crosstalk between the MC3T3-E1 and bEnd.3 cells. Then, we constructed an osteogenesis–angiogenesis coupling chip based on microfluidic technology with channels separated by porous membranes for the indirect co-culture of cells. A dynamic microenvironment of osteogenesis–angiogenesis coupling was established by cyclic microfluidic perfusion, simulating the physiological level of fluid shear stress in vivo (Figure 5). After 14 d of co-culture on the chip, the formation of a mineralized matrix of osteoblasts indicated that the chip can accelerate the process of osteogenesis to improve the effect of osteogenesis (Figure 6). After 3 days of co-culture on the chip, the up-regulation of the angiogenic marker suggested that the chip enhanced the angiogenic effect of endothelial cells (Figure 7). We also found higher levels of VEGF and BMP-2 secretion in the chip model compared to the static co-culture model (Figure 7F,G). These results illustrated that the microfluidic chip enhanced the potential for VEGF- and BMP-2-mediated osteogenesis–angiogenesis coupling (Figure 8).

Interestingly, we found that in the 2:1 (MC3T3-E1:bEnd.3) co-culture group, which exerted an optimal angiogenic advantage, the expression of angiogenic markers in the bEnd.3 cells was lower on day 7 than on day 3 (Figure 4E,F). However, the MC3T3-E1 cells in the co-culture group at this ratio had higher ALP activity than the monoculture group at day 7 (Figure 1C,D), as well as ARS quantification at day 14 and day 21 (Figure 1E–H). This phenomenon suggests that even with optimal cell ratio co-culture, angiogenesis is mainly active in the pre-culture phase to establish a microenvironment that supports subsequent osteogenesis, and the osteogenic activity is intensify with a prolonged co-culture time for continuing differentiation and mineralization. Meanwhile, we propose that the nutrient competition at high cell densities may reduce the expression of angiogenic markers at day 7 in this cell ratio. In conclusion, this result suggests that the relative advantage of osteogenesis–angiogenesis coupling is time-dependent, which is consistent with the physiological process of the fracture healing phases in which the rates and intensities of osteogenesis and angiogenesis differ [48,49,50].

Cell crosstalk in indirect co-culture models occurs mainly through paracrine mechanisms [51]. Meanwhile, cytokines, growth factors, chemokines and other effectors play a critical role in regulating cellular functions [52,53]. Notably, we found that both MC3T3-E1 and bEnd.3 cells secreted VEGF and that the co-culture conditions significantly enhanced VEGF secretion (Figure 7F). This result suggests that osteoblasts can secrete VEGF and mediate angiogenesis in bEnd.3 cells via a paracrine mechanism. This is consistent with the notion that VEGF secreted by MSCs mediates the differentiation of endothelial progenitor cells into ECs via a paracrine mechanism [22]. Some studies have also reported that osteoblast-derived VEGF can regulate osteoblast differentiation and bone formation during bone repair [11]. Therefore, it was hypothesized that VEGF secreted by MC3TE-E1 can regulate its own osteogenic differentiation.

In addition to exploring the role of VEGF in cellular crosstalk, BMP-2 deserves to be studied in depth. It was found that BMP-2 secretion could not be detected in the monocultured bEnd.3 cells; however, the BMP-2 detected in the co-culture was higher than in the monocultured MC3T3-E1 cells (Figure 7G), suggesting that the co-culture conditions enhanced the secretion of BMP-2. Therefore, we hypothesized that MC3T3-E1 cells would be able to promote BMP-2 secretion from bEnd.3 cells, and that bEnd.3 cells would increase the expression of BMP-2 by MC3T3-E1 cells, thereby contributing to the increase in BMP-2 in the co-culture system. Considering the osteogenic effects of VEGF on osteoblasts [54,55], we speculated that BMP-2 also promotes angiogenesis in endothelial cells. The proliferation, migration and immunofluorescence staining suggested that BMP-2 positively promotes angiogenesis in endothelial cells (Figure S6A–F). The discovery of the BMPR2 in the bEnd.3 cells (Figure S6G) further confirmed that the binding of BMP-2 to its receptor affects the function of angiogenesis in bEnd.3 cells. The enhanced BMPR2 expression in the bEnd.3 cells under co-culture conditions (Figure S6H,I) further verified that co-culture enhanced BMP-2-mediated angiogenesis in the endothelial cells. Combining the dual roles of VEGF and BMP-2 in promoting osteogenesis and angiogenesis [56,57], it is hypothesized that VEGF and BMP-2 mediate the bidirectional regulation of osteogenesis and angiogenesis in the co-culture model.

Current mechanistic explanations are mainly based on static culture systems, which may overlook the promotion of paracrine factor secretion by mechanical stimuli. In this study, we demonstrated a beneficial effect of mechanical stimulation on osteogenesis–angiogenesis coupling mediated by paracrine mechanisms using microfluidic chip modeling (Figure 6 and Figure 7). This finding is consistent with mechanical stimulation spatially coupled precursor osteoblast mobilization to angiogenesis via Yes-associated protein and transcriptional co-activator with PDZ-binding motif, regulating vascular morphogenesis to control cartilage remodeling [58]. Furthermore, it is consistent with the notion that fluid shear stress can modulate vascular endothelial cell structure and function [59,60]. Mechanical stimulation can affect cellular function by promoting cytokine secretion. For example, Grit Kasper et al. showed that conditioned medium from mechanically stressed MSCs promoted angiogenesis in human dermal microvascular endothelial cells and attributed these findings to a significant increase in the levels of matrix metalloproteinase-2, transforming growth factor-beta and fibroblast growth factor [61]. Gardner et al. demonstrated increased secretion of soluble factors such as VEGF and matrix metalloproteinase-13 following induction of chondrogenesis by multiaxial mechanical loading [62]. In this study, the VEGF and BMP-2 secretion was increased in the chip compared to the static co-culture. This suggests that physiological levels of fluid shear stress enhanced the VEGF- and BMP-2-mediated crosstalk between osteoblasts and vascular endothelial cells.

In addition to influencing paracrine factors, the direct effects of mechanical stimulation on cell morphology and function are worth considering. The cytoskeleton of both cells in the chip was parallel to the microfluidic direction (Figure 6E and Figure 7C), which indicates that osteoblasts and endothelial cells receive and conduct mechanical signals through the cytoskeleton and respond to mechanical signals through cytoskeleton reorganization [23,41]. This result is consistent with the idea that the cytoskeleton not only supports cell shape but also influences osteoblast function through mechanosensing and signaling, which extended this result to multicellular systems [41,63]. Based on the results of the enhanced coupling potential of the microfluidic chips, it is further hypothesized that altered cell morphology affects the expression of osteogenesis- and angiogenesis-related proteins, facilitates cellular crosstalk, and accelerates the process of tissue formation.

In conclusion, the microfluidic chip enhanced the potential of osteogenesis–angiogenesis coupling mediated by microfluidic perfusion and paracrine signaling, further exploiting the advantages of organ-on-a-chip in the study of complex tissue interactions. However, our research needs to be further developed. For example, the osteogenesis–angiogenesis coupling models involved fewer cell types. Macrophages also play an important role in this process and could be included in the model to further improve the physiological relevance. In addition, the dual-channel design of the chip allows for a smaller number of cells to be harvested, which is a limitation for gene-level detection, and a multi-channel design could be considered in the future. Moreover, the specific signaling pathways of osteogenesis and angiogenesis that may be affected by microfluidic stimulation are needed to improve the understanding of osteogenesis–angiogenesis coupling under mechanical stimulation.

## 5. Conclusions

In this study, we established dynamic and static models of osteogenesis–angiogenesis coupling and compared the effects of coupling within the two models. In the static Transwell insert model, the MCET3-E1 and bEnd.3 cells exhibited a ratio-dependent reciprocal relation and exerted osteogenic and angiogenic advantages under co-culture conditions with different cell ratios. Then, an organ-on-a-chip model mimicking osteogenesis–angiogenesis coupling based on microfluidic technology was established. The microfluidic chips enhanced the potential for VEGF- and BMP-2-mediated osteogenesis–angiogenesis coupling. Our results demonstrated that the microfluidic stimulation induced the directional rearrangement of the MC3T3-E1 and bEnd.3 cytoskeleton, revealing a positive role of the cytoskeleton in mechanotransduction. In a word, the microfluidic chip enhanced the potential for mechanical force- and paracrine-mediated osteogenesis–angiogenesis coupling. This model opens up new avenues for studying the dynamic interplay between osteogenesis and angiogenesis processes in a physiologically relevant microenvironment, which is critical for advancing bone tissue engineering.

## Data Availability

Dataset available on request from the authors.

## Supplementary Materials

The following supporting information can be downloaded at https://www.mdpi.com/article/10.3390/mi16050539/s1, Figure S1: The design of th e chip. (A) Structure diagram of the PDMS chip, including PDMS upper substrate, PET membrane and PDMS lower substrate. The upper and lower substrates are each designed with a channel that can cultivate a type of cell. (B) Housing mounting frame consisting of upper frame and lower frame with observation chamber. (C) PMMA mold, including A-side and B-side, corresponding to the lower and upper substrate of the chip, respectively. The mold can make six chips at a time by the molding method. Figure S2: The microfluidic platform. Figure S3. Mixed medium has no significant effect on osteoblasts and endothelial cells. (A) Effect of 1:1 (MC3T3-E1:bEnd.3) mixed media on the proliferation of MC3T3-E1. (B) Effect of 2:1 (MC3T3-E1:bEnd.3) mixed media on the proliferation of bEnd.3. 1:0: monoculture, 1:1: MC3T3-E1:bEnd.3, 2:1: MC3T3-E1:bEnd.3. Figure S4. 1:1 Co-culture increased the expression of angiogenesis-related markers in MC3T3-E1. Figure S5. Osteoblast and vascular endothelial cell status on chip at different time points. Figure S6. BMP-2 promoted angiogenesis in bEnd.3 cells. (A) Effect of BMP-2 on the proliferation of bEnd.3. (B) Effect of 500 ng/mL BMP-2 on bEnd.3 Transwell migration. (C) Quantification of Transwell migration. (D) Effect of 500 ng/mL BMP-2 on scratch wound assay. (E) Quantification of the scratch wound assay. (F) Expression of CD31 under different culture conditions. (G) Expression of BMPR2 under different culture conditions. (H) Quantification of CD31. (I) Quantification of BMPR2. Table S1. Details of primer sequences used for RT-qPCR analysis of osteogenic differentiation. Table S2. Details of primer sequences used for RT-qPCR analysis of angiogenesis. Table S3. Details regarding the antibodies used in this work.

## Abbreviations

The following abbreviations are used in this manuscript:

VEGF	Vascular endothelial growth factor
BMP-2	Bone morphogenetic protein-2
HIF-1α	Hypoxia-inducible factor-1α
ECs	Endothelial cells
Elisa	Enzyme-linked immunosorbent assay
ARS	Alizarin Red S
MSCs	Mesenchymal stem cells
PDMS	Polydimethylsiloxane
PET	Polyester
PMMA	Polymethyl methacrylate
CCK-8	Cell Counting Kit-8
ALP	Alkaline phosphatase
VEGF-A	Vascular endothelial growth factor A
VEGFR1	Vascular endothelial growth factor receptor 1
VEGFR2	Vascular endothelial growth factor receptor 2
COL1	Collagen type I
RT-qPCR	Quantitative reverse transcription polymerase chain reaction
BMPR2	Bone morphogenetic protein receptor type 2
Runx2	Runt-related transcription factor 2
CD31	Platelet endothelial cell adhesion molecule-1
FITC	Fluorescein isothiocyanate
DAPI	4′,6-diamidino-2′-phenylindole
OPN	Osteopontin
F-actin	Filamentous actin
OCN	Osteocalcin
TGF-β	Transforming growth factor-beta
