# Supplementary material for: Cell Ratio-Dependent Osteoblast–Endothelial Cell Crosstalk Promoting Osteogenesis–Angiogenesis Coupling via Regulation of Microfluidic Perfusion and Paracrine Signaling
Source: Micromachines (Basel). 2025 Apr 30;16(5):539. doi: 10.3390/mi16050539 (PMC12114372; doi:10.3390/mi16050539)
Supplement: Supplementary file 1 [file micromachines-16-00539-s001.zip › micromachines-3586806-supplementary.pdf]

Supplementary Materials

Cell Ratio-Dependent Osteoblast–Endothelial Cell Crosstalk Promoting Osteogenesis–Angiogenesis Coupling via Regulation of Microfluidic Perfusion and Paracrine Signaling

Yuexin Wang, Shu Chen, Wenwen Fan, Sixian Zhang and Xi Chen *

Key Laboratory for Ultrafine Materials of Ministry of Education, Frontiers Science Center for Materiobiology and Dynamic Chemistry, Engineering Research Center for Biomedical Materials of Ministry of Education, School of Materials Science and Engineering, East China University of Science and Technology,
Shanghai 200237, China
* Correspondence: chenxi@ecust.edu.cn; Tel./Fax: +86-21-64251308

SUPPLEMENTARY METHODS

Device development

The microfluidic system (Figure S2) consists of an inlet sump, a peristaltic pump (Masterflex® Ismatec® IPC, Avantor), a chip and a reservoir. The peristaltic pump is connected via an adapter tube to a 0.25 mm ID catheter (PharMed® BPT Tubing, Masterflex), which in turn is connected via an adapter tube to a 0.56 mm ID polyethylene catheter, which is then connected to the entire device. The chip is attached to the catheter via a 0.5 mm ID steel pin to facilitate fluid flow entry and exit. The entire de-vice is maintained in a carbon dioxide incubator (37 °C, 5% CO_2) for 2 weeks.

A. SUPPLEMENTARY FIGURES

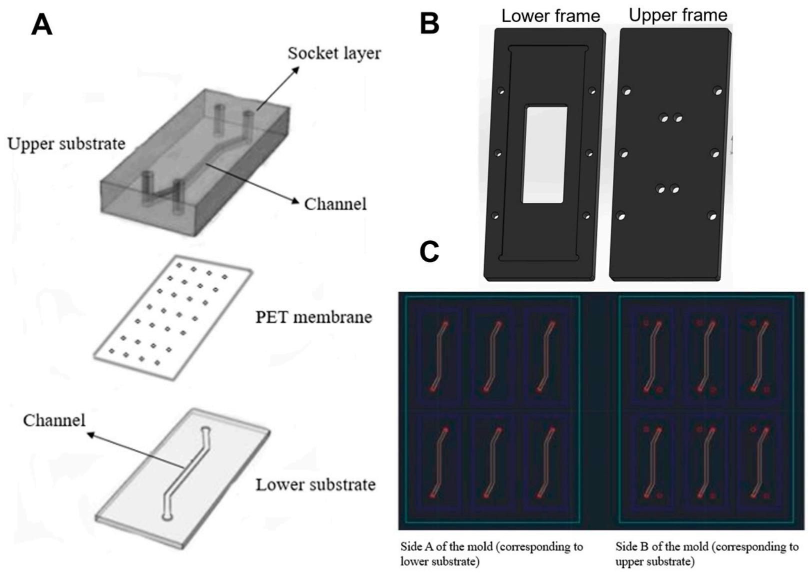

Figure S1. The design of the chip. (A) Structure diagram of the PDMS chip, including PDMS upper substrate, PET membrane and PDMS lower substrate. The upper and lower substrates are each designed with a channel that can cultivate a type of cell. (B) Housing mounting frame consisting of upper frame and lower frame with observation chamber. (C) PMMA mold, including A-side and B-side, corresponding to the lower and upper substrate of the chip, respectively. The mold can make six chips at a time by the molding method.

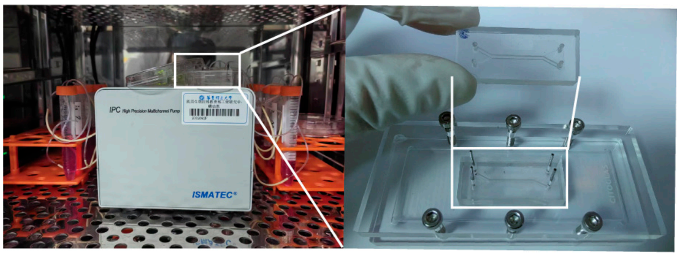

Figure S2. The microfluidic platform.

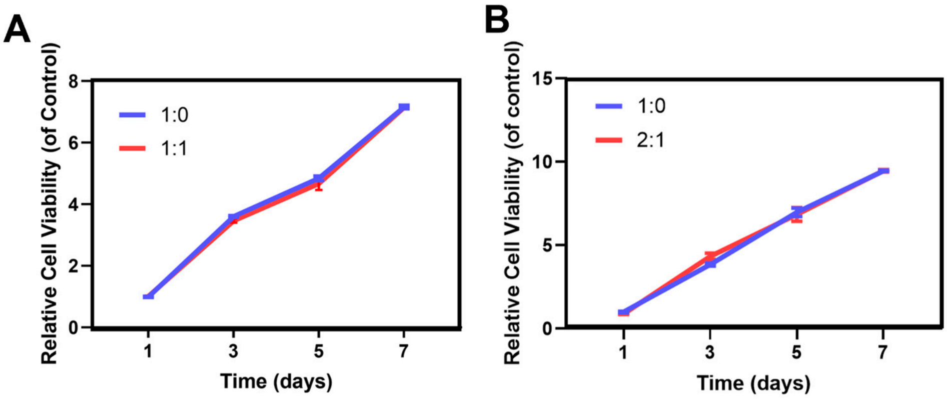

Figure S3. Mixed medium has no significant effect on osteoblasts and endothelial cells. (A) Effect of 1:1 (MC3T3-E1:bEnd.3) mixed media on the proliferation of MC3T3-E1. (B) Effect of 2:1 (MC3T3-E1:bEnd.3) mixed media on the proliferation of bEnd.3. 1:0: monoculture, 1:1: MC3T3-E1:bEnd.3, 2:1: MC3T3-E1:bEnd.3.

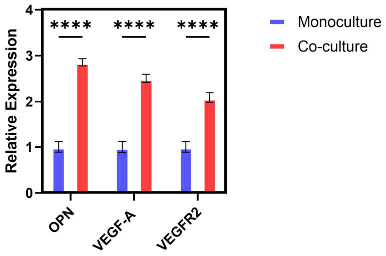

Figure S4. 1:1 Co-culture increased the expression of angiogenesis-related markers in MC3T3-E1.

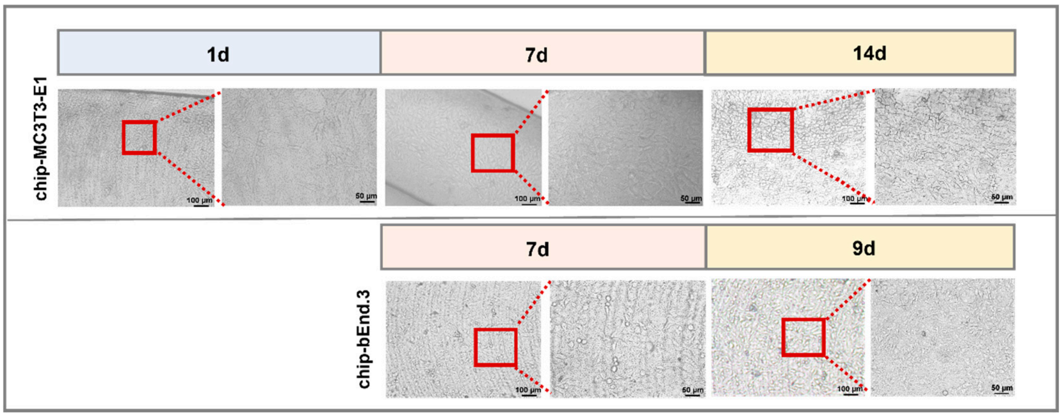

Figure S5. Osteoblast and vascular endothelial cell status on chip at different time points.

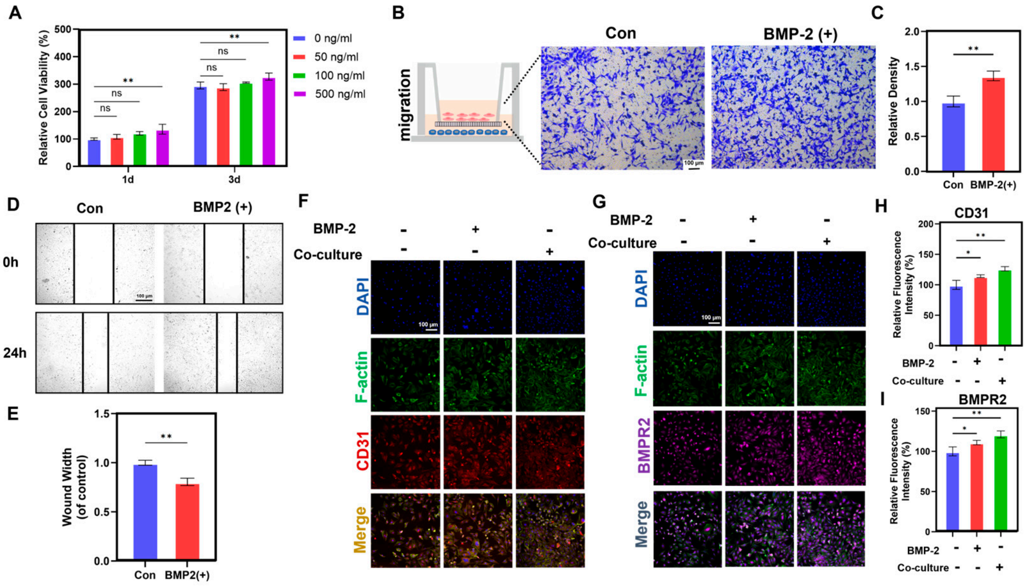

Figure S6. BMP-2 promoted angiogenesis in bEnd.3 cells. (A) Effect of BMP-2 on the proliferation of bEnd.3. (B) Effect of 500 ng/mL BMP-2 on bEnd.3 Transwell migration. (C) Quantification of Transwell migration. (D) Effect of 500 ng/mL BMP-2 on scratch wound assay. (E) Quantification of the scratch wound assay. (F) Expression of CD31 under different culture conditions. (G) Expression of BMPR2 under different culture conditions. (H) Quantification of CD31. (I) Quantification of BMPR2.

Table S1. Details of primer sequences used for RT-qPCR analysis of osteogenic differentiation.

Gene	Forward primer (5'-3')	Reverse primers (5'-3')
ALP	AAGCTGGGAAGAACACTCCA	CAAACAGGAGAGCCACTTCA
COL 1	TGACTGGAAGAGCGGAGAGT	GACGGCTGAGTAGGGAACAC
Runx2	CCTCTGACTTCTGCCTCTGG	ATGAAATGCTTGGGAACTGC
BMPR2	CAGCTGGCCAGGCAGCCAAC	TGGCCAGCCTGTTGCTCTCG
OPN	CATTCTCGGAGGAAACCAGC	GAATTCAGCCAGGAGAACTGC
OCN	TTTCTGCTCACTCTGCTGACC	ATGCGTTTGTAGGCGGTCTT
BMP-2	TTCCATCACGAAGAAGCCGT	GTCGAAGCTCTCCCACTGAC
GAPDH	TGAACGGGAAGCTCACTGG	GCTTCACCACCTTCTTGATGTC

Table S2. Details of primer sequences used for RT-qPCR analysis of angiogenesis.

Gene	Forward primer (5'-3')	Reverse primers (5'-3')
VEGFR1	GTCTCCATCAGTGGCTCTACG	CCCGGTTCTTGTTGTATTTTG
VEGFR2	CTGCCTACCTCACCTGTTTCC	CGGCTCTTTCGCTTACTGTTC
VEGF	ACACGGGAGACAATGGGATGA	CAGGGCCAGGAATGGGTTTG
CD31	GCAACTATTAAGGTGGCGATG	CAGAGCCAGCAGTATGAGGAC
HIF-1α	ACCTTCATCGGAAACTCCAAAG	CTGTTAGGCTGGGAAAAGTTAGG
VEGF-A	CAAACCTCACCAAAGCCAGC	TTAACTCAAGCTGCCTCGCC
GAPDH	TGTGTCCGTCGTGGATCTGA	TTGCTGTTGAAGTCGCAGGAG

Table S3. Details regarding the antibodies used in this work.

Antibody	Manufacturer	Catalog No.	Dilution
Rabbit Anti-VEGFR2 pAb	Proteintech, Wuhan, China	26415-1-AP	1:400
Rabbit Anti-Runx2 pAb	Servicebio, Wuhan, China	GB115631	1:400
Rabbit Anti-BMP-2 pAb	Servicebio, Wuhan, China	GB11252	1:400
Mouse Anti-OPN mAb	Servicebio, Wuhan, China	B122328	1:400
Mouse Anti- COL1 mAb	Servicebio, Wuhan, China	GB154197	1:400
Rabbit Anti-OCN pAb	Proteintech, Wuhan, China	23418-1-AP	1:400
Rabbit Anti-BMPR2 pAb	Proteintech, Wuhan, China	14376-1-AP	1:400
Mouse Anti-CD31 mAb	Proteintech, Wuhan, China	GB15063	1:400
Alexa Fluor 488-conjugated Donkey Anti-goat IgG (H+L)	Abcam, Cambridge, England	ab150129	1:1000
Alexa Fluor 568-conjugated Donkey Anti-rat IgG (H+L)	Abcam, Cambridge, England	ab175475	1:1000
Alexa Fluor 647-conjugated Donkey Anti-rabbit IgG (H+L)	Abcam, Cambridge, England	ab150075	1:1000
